# Body composition indices in a sample of female adolescents with postural deformity: a case control study

**DOI:** 10.1186/s13104-019-4794-y

**Published:** 2019-11-20

**Authors:** Dina Golalizadeh, Vahideh Toopchizadeh, Negar Fasaie, Neda Dolatkhah

**Affiliations:** 1 Faculty of Medicine, Tabriz University of Medical Science, Tabriz, Iran; 2 Physical Medicine and Rehabilitation Research Center, Aging Research Institute, Tabriz University of Medical Science, Tabriz, Iran

**Keywords:** Body composition, Adolescent, Posture

## Abstract

**Objectives:**

Normal posture is considered to be an indicative of good musculoskeletal health in school aged adolescents. Little is known about the body composition indices in relation with postural deformities in adolescents. The aim of this study was to assess relation of body composition analysis indices with postural deformities in a sample of female high school adolescents.

**Results:**

In this case–control study, 37 eligible female adolescents with any postural deformities and 33 normal posture subjects enrolled in the study by random cluster sampling. Body composition analysis performed by bioelectrical impedance analysis (BIA) method to quantity body fat mass (BFM), soft lean mass (SLM) and lean body mass (LBM). The binary logistic regressions were performed to evaluate the associations of body composition indices which were significantly different between two groups at significance level of 0.05 with postural disorders in the two groups. There was no significant difference between subjects with postural disorders with their normal controls concerning demographic variables. We observed an inverse association between postural deformity risk and LBM (OR = 0.803; 95% CI 0.690–0.934) and SLM (OR = 0.774; 95% CI 0.649–0.922) after adjusting the analysis by height of participants.

## Introduction

Faulty posture in school-age children and teens is one of the most common and yet underestimated health disorders [1, 2]. Enlarged incidence of discordant posture and spinal deformities in children is endorsed by investigators from several countries and is considered as a pandemic of current decades [3]. Some postural deformities are representative of human growth and development; however others are detrimental and can disturb the life quality [4]. A review of studies in this area shows that the prevalence of postural deformities is also high in Iran [5–7]. Many factors such as age, weight, body mass index (BMI), gender, and race can impress the musculoskeletal system [8].

Body composition and anthropometric indices are probable to shape children sagittal upright posture [9–12]. Their special effects are evidently associated with the normal development of the musculoskeletal system in childhood, with fat mass leading to plastic variations in all structures such as muscles and bones that prevail during the life period [9–12].

Due to the increasing prevalence of obesity and overweight in adolescence and children, the association of body composition and anthropometric indices with occurrence of these disorders is yet an open question [13–15]. On the other hand, the comparison of anthropometric and body composition indices in adolescents with postural disorders with normal posture controls has never been performed, and it is unidentified whether one or more specific body composition parameters more contribute in sagittal postural deformities. This study aimed to determine the relationship between body composition’s components and postural disorder in a sample of Iranian female adolescents.

## Main text

### Methods

#### Participants

This case–control study was conducted among female high school students in Tabriz, Iran. A trained medical student referred to high schools educational organization of Tabriz city, Iran and selected four girls’ high schools by random cluster sampling. Then she went to selected high schools and listed the students and selected 100 students per school by random sampling. Selected students and their parents invited to a briefing session and the objectives and methods of the study were fully explained to them and informed consent obtained from students and their parents. Fifty eligible participants with postural disorder enrolled in the case group during a period of 4 months from September to December 2018. For each case, one control was selected from the same school. Inclusion criteria were as follows: ages ranging from 14 to 18 years old, willingness to participate in the study. Finally data of 37 adolescent with postural disorder and 33 healthy controls were analyzed.

#### Postural examination

A trained physical Medicine and Rehabilitation resident went to the schools and complete physical examinations were done by her for all selected students. Students were asked to dress up and take off their shoes and then standing in normal position against the checkered plate mounted the wall. For assessing of shoulder asymmetry, each student viewed anterior and posteriorly in front of checkered plate. A scoliometer was applied for screening of scoliosis in this study and deviation of more than 5° was considered as scoliosis [16, 17]. For screening of the thoracic kyphosis in adolescence, Debrunner kyphometer was used. In this method, kyphometer stands were placed on the 4th and 12th thoracic vertebra and the angle more than 44° was noted as kyphosis disorder [18, 19]. For determination of lumbar lordosis, a flexible ruler was used which have high validity and reliability. In this technique, researcher asked the students to open their feet about 10 to 15 cm and the flexible ruler placed on the spinous process of the T12 to the spinous process of S2 vertebra and shaped then transferred to a paper, Arc angle was calculated with this formula:

$${\text{Arc}}\tan \left( {2{\text{H}}/{\text{L}}} \right)4\, =\uptheta\text{.}$$

The angles greater than 40° was considered hyperlordosis [20, 21].

In order to, for diagnose of the genuvalogum and genovarum, the students were asked to stand upright and respectively the interval between the inner malleolus and the intercondylar space were measured. Each space more than 6 cm were considered as abnormal [22].

#### Anthropometric measurements

Body weight was measured, without shoes and weighty clothing by a digital scale (Seca, Hamburg, Germany) to the nearest 0.1 kg. Height without shoes was measured by a non-stretched tape measure (Seca, Hamburg, Germany) to the nearest 0.1 cm. To assessment of anthropometric measurements, body mass index (BMI) was calculated according to the formula [3]:

$${\text{BMI}} = {\text{body mass expressed in kg}}/\left( {\text{body height}} \right) 2 {\text{ expressed in meters}} .$$

#### Body composition analysis

One of the most suitable methods for measuring anthropometric indices is the use of a fast and non-invasive method of bioelectrical impedance analysis (BIA) to quantity body ratios which, with more precision and relatively less cost (InBody270). Body composition, the calibration of body fat in association with LBM is computed from the variance in conductance because fat-free body mass represents negligible impedance to electrical current due to a great value of water and electrolytes while fat mass displays very little conductance to electrical signals [23]. Body fat mass (BFM) refers to the total amount of fats that can be extracted from fat and other cells. BFM is calculated by excluding LBM from body weight. Soft lean mass (SLM) can be calculated by excluding the mineral found in the bones from LBM.

#### Physical activity

Physical activity of the participants was assessed by the short-form IPAQ (International Physical Activity Questionnaire) [24]. Three categories of physical activity were suggested: low, moderate, and high [25]. This questionnaire has been already validated for application in Iran [26].

#### Sample size and statistical analysis

Sample size was based on a pilot study designed on nine students with postural disorder and nine students without any disorder, with the following criteria: the mean of BMI in children with and without postural disorder (20.30 ± 2.34 and 22.40 ± 3.38, respectively) and the minimum sample size of 31 persons in each group was calculated with α = 0.05 with 95% confidence interval and 80% test power.

All statistical analyses were done by Statistical Package for the Social Sciences (SPSS v.22 for Windows, SPSS Inc, Chicago, IL, USA). Independent sample t-tests and χ^2^-tests were used to evaluate for differences in age, anthropometry and body composition indices between the cases and controls. The relationship between the variables was analyzed using the Pearson’s correlation. The binary logistic regressions were performed to evaluate the associations of body composition indices which were significantly different between two groups at significance level of 0.05 with postural disorders in the two groups adjusting for confounders. Odds ratios (ORs) and 95% confidence intervals (95% CI) for risk of postural disorders were calculated.

### Results

In this study 37 adolescent with postural disorder (case group) and 33 normal adolescent (control group) were studied. Of the 37 participants in case group, 8 patients (21.6%) had scoliosis, 7 patients (18.9%) had kyphosis, 8 patients (21.6%) had lordosis, 20 persons (54.1%) had asymmetric shoulder, 7 patients (18.9%) had genuvarum, 10 patients (27.0%) had genuvalgum and 1 patient (2.7%) had flat-foot.

Table 1 shows demographic characteristics of participants in both groups of study. There was no significant difference between subjects with postural disorders with their normal controls concerning demographic variables.

**Table 1:** Demographic characteristic of participants in case (n = 37) and control (n = 33) groups

Variable	Case	Control	p-value*
Age (year)	16.27 ± 0.87	16.45 ± 0.83	0.369
Study table			
Yes	6 (16.2%)	10 (30.3%)	0.164
No	31 (83.8%)	23 (69.7%)	
Type of bag			
Shoulder bags	8 (21.6%)	2 (6.1%)	0.065
Backpack	29 (78.4%)	31 (93.9%)	
Physical activity			
Low	23 (62.2%)	14 (42.4%)	0.138
Moderate	6 (16.2%)	9 (27.3%)	
High	8 (21.6%)	10 (30.3%)	
Study (h/day)	3.51 ± 1.77	3.33 ± 2.07	0.699
Social media (h/day)	2.56 ± 2.37	2.57 ± 2.19	0.988

Data are presented as Mean ± SD or n (%)

*Obtained from independent samples T test or Chi-Square Test

Table 2 shows anthropometric characteristics and body composition indices of participants in both groups of study. As seen, there were significant differences in terms of height, LBM and SLM between two groups. There was no significant difference in mean weight, BMI and TBF between two groups. The results of within-group analysis showed that there was significant association between BFM and LBM (r = 0.720, p < 0.001), BFM and SLM (r = 0.678, p < 0.001), BFM and minerals (r = 0.895, p < 0.11) and SLM and minerals (r = 0.878, p < 0.001) in subjects with postural disorders. There was also significant association between BFM and LBM (r = 0.818, p < 0.001), BFM and SLM (r = 0.772, p < 0.001), BFM and minerals (r = 0.505, p = 0.003), height and LBM (r = 0.510, p = 0.002) and height and SLM (r = 0.530, p = 0.002) in healthy participants.

**Table 2:** Anthropometric characteristics and body composition indices of participants in case (n = 37) and control (n = 33) groups

Variable	Case	Control	p-value*
Weight (kg)	54.35 ± 11.20	53.98 ± 9.04	0.880
Height (cm)	166.97 ± 5.20	164.00 ± 4.35	0.011
BMI (kg/m^2^)	21.03 ± 4.44	20.60 ± 3.29	0.646
BFM (kg)	13.79 ± 8.03	11.13 ± 5.29	0.103
LBM (kg)	40.55 ± 3.92	42.85 ± 4.18	0.021
SLM (kg)	37.44 ± 3.41	39.39 ± 3.66	0.025
Minerals	3.11 ± 0.57	3.46 ± 1.16	0.109

Data are presented as Mean ± SD or n (%)

*Obtained from independent samples T test or Chi-Square Test

To determine the odds ratio (OR) of postural disorder risk factors in adolescents, multiple logistic regression was used. The results are presented as Table 3.

**Table 3:** Multiple logistic regression analysis of postural disorder predictors

Variable	Crude OR (95% CI)	p-value	Adjusted OR^a^ (95% CI)	p-value
SLM	0.845 (0.723–0.988)	0.035	0.774 (0.649–0.922)	0.004
LBM	0.859 (0.749–0.986)	0.030	0.803 (0.690–0.934)	0.005

^a^OR adjusted for height

### Discussion

According to the results of the study, there was no significant difference between the participants with postural deformity compared with adolescents without deformity except LBM and SLM. The LBM and SLM had protective effects against postural disorders in adolescents. A 1-standard deviation increase in SLM was associated with lower odds of postural disorders (0.774, 95% CI 0.64–0.92).

In the modern civilized world of expansion of mechanization and electronics, children and adolescents from the earliest years spend free time choosing sitting position (in front of TV/computer). Lacking physical activity leads to muscular misbalance, which can be a risk factor for disorders in body posture [27, 28]. On the other hand the continual bearing of heavy school bags imposes additional tension on the developing spine of children, making it more disposed to postural deviations and finally back complications [29, 30].

Essentially, body posture is based on the interaction of kinesthetic sense, muscle balance and neuromuscular harmony. The fat and fat-free masses operate as extra-skeletal regulators of bone morphology. Adiposity and muscles can directly affect posture by altering the direction of vertebral bodies [10, 31, 32]. Therefore, mechanical loading of the skeleton by fat and fat-free tissues seems to be a critical factor and improve postural health in children [9, 11].

We are unaware of any previous reports from the association between LBM and body posture of adolescents in the available literature. Nevertheless, there are some reports of the association between composition of body mass and foot postural disorders [33, 34].

Cross-sectional and longitudinal studies have shown that postural disorders, even in normal weight children, are related with adiposity increases [35, 36]. It has been confirmed that children with normal BMI may have a higher content of fat in their body with lesser lean muscle mass [37]. Additionally, results of cross-sectional studies with various age groups suggest weight through growth to be an issue in standing posture evolvement [2, 38]. Inordinate body mass in obese/overweight children may originate a decline in the consistency and the necessity to look for postural mechanisms of accommodation. This can originate alterations in usual balance alignment causing in extended lumbar lordosis of abdominal protrusion and pelvic anteversion. On the contrary, a longitudinal study of 11 to 22-year-old participants established no association between BMI and the appearance of thoracic kyphosis [39].

Our finding that height confounded the association of lean mass with posture is not unexpected. Up-right spinal posture evolvement may be impressed by height [2], gender [39, 40], exercise levels [41] and school equipment [42]. Exercise levels have been stated to affect the evolvement of posture [41]. Lower physical activity may lead to obesity and muscle deconditioning, and then affecting postural status.

### Conclusion

There were significant differences concerning body composition indices containing LBM and SLM in female adolescents with any postural deformities in comparison with normal posture ones. These indices (LBM and SLM) are protective from postural deformities in female normal weight adolescents.

## Limitations

Limitation of this study is that it is a case–control study carried out with a small sample size, thus more extensive with improved design studies are suggested to be performed, considering the family history, with data about pathological disorders such as joint hypermobility. Secondly, diagnosis of the postural deformities was limited to visual analysis. At present time, there are some new computerized diagnostic methods that are used in clinical practice. In addition, selecting female participants raises the question of generalizability the results to all adolescents.

## Data Availability

All the necessary data are presented herewith. However if needed, raw data on excel format can be availed on reasonable request from the corresponding author.

## Abbreviations

BFM: body fat mass
BIA: bio impedance analysis
BMI: body mass index
CI: confidence intervals
IPAQ: International Physical Activity Questionnaire
LBM: lean body mass
OR: odds ratio
SLM: soft lean mass
